# Influences on eating: a qualitative study of adolescents in a periurban area in Lima, Peru

**DOI:** 10.1186/s12889-016-2724-7

**Published:** 2016-01-15

**Authors:** Jinan C. Banna, Opal Vanessa Buchthal, Treena Delormier, Hilary M. Creed-Kanashiro, Mary E. Penny

**Affiliations:** 1 Department of Human Nutrition, Food, and Animal Sciences, College of Tropical Agriculture and Human Resources, Agricultural Sciences 216, University of Hawaii at Manoa, 1955 East–west Road, Honolulu, HI 96822 USA; 2 Office of Public Health Studies, University of Hawaii at Manoa, 1960 East–west Road, Honolulu, HI 96822 USA; 3 Instituto de Investigación Nutricional, Av. La Molina 1885, Lima 12, Peru

**Keywords:** Child nutrition sciences, Adolescent, Qualitative research, Peru, Latin America

## Abstract

**Background:**

Peruvian adolescents are at high nutritional risk, facing issues such as overweight and obesity, anemia, and pregnancy during a period of development. Research seeking to understand contextual factors that influence eating habits to inform the development of public health interventions is lacking in this population. This study aimed to understand socio-cultural influences on eating among adolescents in periurban Lima, Peru using qualitative methods.

**Methods:**

Semi-structured interviews and pile sort activities were conducted with 14 adolescents 15–17 years. The interview was designed to elicit information on influences on eating habits at four levels: individual (intrapersonal), social environmental (interpersonal), physical environmental (community settings), and macrosystem (societal). The pile sort activity required adolescents to place cards with food images into groups and then to describe the characteristics of the foods placed in each group. Content analysis was used to identify predominant themes of influencing factors in interviews. Multidimensional scaling and hierarchical clustering analysis was completed with pile sort data.

**Results:**

Individual influences on behavior included lack of financial resources to purchase food and concerns about body image. Nutrition-related knowledge also played a role; participants noted the importance of foods such as beans for anemia prevention. At the social environmental level, parents promoted healthy eating by providing advice on food selection and home-cooked meals. The physical environment also influenced intake, with foods available in schools being predominantly low-nutrient energy-dense. Macrosystem influences were evident, as adolescents used the Internet for nutrition information, which they viewed as credible.

**Conclusions:**

To address nutrition-related issues such as obesity and iron-deficiency anemia in Peruvian adolescents, further research is warranted to elucidate the roles of certain factors shaping behavior, particularly that of family, cited numerous times as having a positive influence. Addressing nutrition-related issues such as obesity and iron-deficiency anemia in this population requires consideration of the effect of social and environmental factors in the context of adolescent lifestyles on behavior. Nutrition education messages for adolescents should consider the cultural perceptions and importance of particular foods, taking into account the diverse factors that influence eating behaviors.

## Background

Adolescence is a time of transition in the lifespan. During this period, the physical, social and developmental changes that take place play an important role in determining eating behaviors and health [1]. Due to an accelerated growth rate and increased caloric needs, adolescence is a nutritionally vulnerable time during which both dietary excess [2] and insufficiency [3, 4] and lack of variety and balance are common [5]. It is also a period during which adolescents gain independence and select their own foods to a greater degree. As more independent choices are made, consumption of low-nutrient energy-dense (LNED) foods may increase, with a consequent excess intake of added sugar and fat, and inadequate intake of micronutrients such as calcium, iron, zinc, and vitamins A and C [6]. The changes that occur during adolescence may contribute to the development of a number of nutrition-related problems that may have lasting effects throughout the lifespan [7].

As part of the development of interventions to address nutritional issues in adolescent populations, it is necessary to understand contextual factors that influence eating habits. Adolescence is a period of susceptibility to a number of influences on diet. Story et al. present a theoretical framework to understand influences on behavior, which includes four levels: individual (intrapersonal), social environmental (interpersonal), physical environmental (community settings), and macrosystem (societal) [1]. Individual influences include food preferences, found to be a strong predictor of food choices [8, 9], and knowledge of why and how to eat healthily, which may or may not translate into healthful behavior [10]. At the interpersonal level, peers play a large role in determining habits [11], as does family [12, 13]. The physical environment also plays a part in influencing behavior, with the school environment being of particular importance. A number of studies in children and adolescents have found positive associations between the availability of LNED foods in schools and calorie intake from these items, as well as body mass index [14–16]. At the societal level are media influences; several recent studies in diverse adolescent populations have demonstrated associations between excessive television and computer use and undesirable eating behaviors that may contribute to overweight and obesity [17, 18].

Low and middle-income countries represent an important area of focus in addressing nutritional issues in adolescents, as there has been a rise in non-communicable diseases in many regions, while malnutrition persists [19–21]. A number of studies have been published examining influences on eating habits among adolescents in low- and middle-income countries to inform the design of interventions to promote desirable health behaviors. In a study of students ages 13 to 15 years from four African countries, for example, Peltzer [22] identified several factors at the interpersonal level that proved to be important for adolescent health, including peer support at school and parental supervision. In a study of Chinese adolescents examining weight-related perceptions and behaviors, Xie et al. identified influences at the societal level impacting behavior, such as exposure to Western media [23]. Researchers have also sought to elucidate the factors influencing health-related habits in low- and middle-income countries, however, previous studies have generally employed quantitative methods that may not account for culture-specific influences on behavior. Research using in-depth qualitative methods is lacking, and may further elucidate the complexity of factors impacting adolescent health behaviors.

Research aiming to identify influences on adolescent behavior within the cultural context is particularly relevant to Peru, a middle income country. Peruvian adolescents are at high nutritional risk, facing issues such as overweight and obesity, anemia, and pregnancy during a period of development. The prevalence of overweight in adolescents 15 to 19 years of age is 19.1 %, and the prevalence of obesity is 4.5 % [24]. In women in this age group, 17.2 % suffer from anemia, and 13.2 % already have at least one child [24]. Women living in areas with high rates of anemia and undernutrition who become pregnant during adolescence are more likely to die or suffer complications during pregnancy and delivery [25]; similarly, overweight adolescents have increased risk of neonatal and perinatal problems [26]. While dietary deficiencies lead to anemia, at the same time dietary excess is leading to increasing overweight and obesity, a double burden of malnutrition that presents a challenge in terms of development of health-related interventions [24, 27]. In addressing the nutritional issues in Peru, interventions that account for the cultural context and culture-specific influences on behavior are needed. The objective of the current study is to gain insight into socio-cultural influences on eating in adolescence by conducting in-depth interviews with adolescents in periurban Lima, Peru.

## Methods

### Participants and recruitment

Subjects were males (*n* = 8) and females (*n* = 6) 15–17 years in Canto Grande, a periurban shantytown community in San Juan de Lurigancho, Lima, Peru, a district in the costal desert typical of the large sprawling densely populated periurban districts surrounding central Lima. This is one of the poorer districts of Lima, in which most adults are employed in informal or low-paying jobs and are classified as having low socioeconomic status [28]. Overweight and obesity are common among adolescents here; in an examination of the weight status of 54 female and 34 male adolescents 13–17 years of age seen at the vaccine clinic at the Instituto de Investigación Nutricional in Canto Grande, a community within the district of San Juan de Lurigancho, 37 % of girls and 32 % of boys were found to be overweight or obese, reflecting higher rates than at the national level (M. Penny, personal communication, January 28, 2015).

Participants were selected using a census of inhabitants in Canto Grande to yield a purposeful random sample. The sample was purposeful in that a specific subgroup of adolescents within the broader adolescent population in Lima was the focus. Adolescents 15–17 years of age living in the selected community were eligible for inclusion. Once those eligible to participate had been identified using the census, researchers selected participants at random, visited the homes of those selected, and invited each to participate. Female subjects who had previously been or were pregnant were excluded, as the aim was to examine influences on typical eating habits in adolescence. The sample size was determined using the concept of saturation of conceptual categories, or the point at which no new information was observed in the data [29]. This was achieved through an iterative process of data collection and review of the data until there was failure to uncover any new concepts in the interviews [30]. The first author constantly checked and rechecked the elemental codes and concepts in a back-and-forth interplay with the data [30]. While there is little evidence in the literature regarding the ideal number of participants to be included in qualitative studies, Guest et al. found in a systematic documentation that saturation occurred within the first 12 interviews, providing further support for the sample size in the current study [31]. Parents provided written consent for adolescents to take part in the study, and the adolescents provided written assent (an affirmative agreement to participate in research). Participants were compensated for their time with a pocket-size calculator. The Research Ethics committee of the Instituto de Investigación Nutricional approved the study.

### Procedures

The first author, who was trained in interviewing and had previous experience in qualitative data collection and analysis in low-income Spanish-speaking populations, collected all data in the Spanish language in participants’ homes. Conducting interviews in this setting allowed participants to show examples of food items mentioned during the interview if needed. A local research assistant accompanied the first author to take notes and tape the sessions.

### Free list of foods and pile sorts

**Fig. 1:**
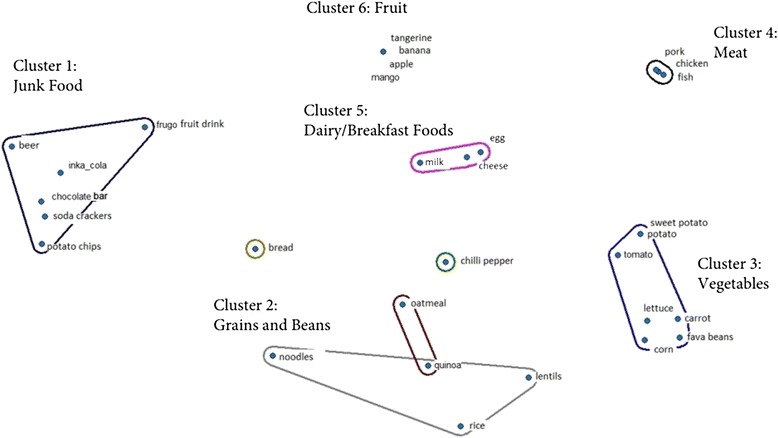
Results of multidimensional scaling and hierarchical clustering analysis using pile sort data from adolescents ages 15–17 years (*n* = 10) in periurban Lima, Peru

Free listing is a semi-structured interviewing method designed to identify the salient items in a defined category from the target population [32]. Four participants (2 male, 2 female) were asked to name all food items consumed in the area, at all times of day, including condiments. In addition, foods included in a pile sort activity in a previous study conducted in Peruvian adolescents in poor periurban communities were examined [33]. Commonly named items were selected to be pictured on a set of cards for use in the pile sort activity, with 14 out of 30 items matching those of the previous study [33]. Examples of items included were rice, beans, and milk (all items shown in the Fig. 1). All food groups were represented in the set.

Pile sorts can be used to stimulate discussion regarding beliefs about food thus allowing further elucidation of influences on eating habits [32], and has been used extensively in research studies [33–36]. Unconstrained pile sorts were conducted with the remaining 10 (6 male, 4 female) participants in the current study. Pile sorting involved giving participants a set of 30 cards, each of which contained a distinct food image, and asking them to ‘pile’ cards into groups. The number of food images include reflect the recommendations of Blum et al., who suggest the use of 25 to 30 food images generated through the administration of the free list [37]. The same number of cards was used in a previous study in Peruvian adolescents, and items from all food groups were included to allow for sufficient items for formation of piles [33]. Participants were asked to group foods perceived to have similar characteristics to form any number of piles. After grouping food cards, participants were asked to name each ‘pile’ and describe the characteristics of the foods placed in that group. In another exercise, they were asked to order the 30 food cards from the most ‘unhealthy’ to ‘healthy,’ and to name any other foods not pictured that they considered ‘healthy’ or ‘unhealthy.’ Numbers written on each card were used to facilitate the recording of data from pile sorts and the ‘ordering’ exercise by hand during the interview.

### Semi-structured interviews

Semi-structured interviews with all participants lasted approximately 60 min. The interview guide was devised by the first author through a review of the literature examining influences on eating in adolescents, and was then reviewed and refined by two co-authors with extensive experience conducting research in the Peruvian population. The guide was designed to elicit information on influences on eating at four levels identified in a previously developed theoretical framework: individual (intrapersonal), social environmental (interpersonal), physical environmental (community settings), and macrosystem (societal) [1]. This was achieved through the use of questions addressing each level of influence on adolescent behavior, with examples of topics addressed at each level as follows: 1) individual: nutrition-related knowledge, including strategies to prevent anemia; 2) social environmental: activities with peers and family; 3) physical environmental: neighborhood and food environment in which eating behaviors occur; 4) macroystem: access to Internet and exposure to media through various types of technology, including cell phones and computers. A limited number of questions related to influences on physical activity habits were also posed (results not reported).

The first author transcribed all audio-recorded interviews with participants verbatim, including the discussion that formed part of the pile sort activity.

### Data analysis

#### Semi-structured interviews

Interview transcripts were analyzed using directed content analysis [38], with the theoretical framework of categories of influence on adolescent eating serving to guide the analysis [1]. Directed content analysis involves using deductive categories to guide the coding of the text. This contrasts with coding that is generated inductively from the data [39]. Beginning with categories from the theoretical framework, the first author identified these key concepts in the data to serve as initial coding categories [40]. With these categories, the first author coded each chunk of text using the NVivo software package version 10 (QSR International Inc., Burlington, MA, USA). Operational definitions were then developed for each category based on the data. The initial coding, including both the coding schema and the actual coded text, was reviewed by two researchers with experience in qualitative analysis. The coding was discussed and refined among the analysis team, with the final coding scheme approved by all three researchers on the team. The process of checking and rechecking codes and concepts allowed for data saturation to be achieved with 14 interviews.

### Pile sorts

Multidimensional scaling, which yields a map of items arranged in proximity to others to reflect groupings, and hierarchical clustering analysis, which produces a refined graphic showing which items were most often grouped together [41], was completed with pile sort data using ANTHROPAC software (Analytic Technologies, Lexington, KY). The data analysis for the qualitative comments participants provided as an explanation for the groupings was conducted using the same steps described above. The descriptions corresponding to each pile and comments about healthy/unhealthy foods were coded according to the aforementioned categories from the theoretical framework. Two co-authors then reviewed and discussed the application of codes to the transcripts with the first author. The coding of the pile sorts categories was validated through review and discussion among the analysis team, using the same protocol as the interview coding.

Validity of the data was ensured using the following strategies. The first was in developing the theory-informed coding scheme to identify the categories and code definitions [40]. The second strategy involved review of the coding scheme and coded transcripts by peer researchers who verified the codes and coding decisions for coherence. During this step codes were revised and agreed upon by the three researchers [40].

## Results

From the pile sort analysis, six main food item clusters were identified to reflect the results of the activity that required participants to group foods into piles according to any system desired. When asked to describe the characteristics of the clusters of food items and assign a name to each group, participants provided the following responses: 1) junk food (non-nutritive, tasty, expensive, chemical); 2) grains and beans (healthy, tasty, lunch foods); 3) vegetables (prevent disease, natural); 4) meat (favorite, fatty, contain hormones); 5) dairy/breakfast foods (consumed daily, contain protein); 6) fruit (prevent disease, contain vitamins). The results of the multidimensional scaling and hierarchical clustering analysis are shown in the Fig. 1. The clusters identified are labeled, with the foods corresponding to each one shown in the Fig. 1.

When asked to rank foods pictured on the cards from least to most healthy, items such as beer and hot pepper were generally placed first, followed by processed foods such as chocolates, soda, and potato chips. ‘Whole’ foods such as fruit, vegetables, beans, meat and dairy were often placed toward the ‘healthy’ end of the list, with ‘quinoa’ ranked as the healthiest in several cases. Foods often mentioned as healthy but not pictured included legumes such as green split peas, whole grains such as *kiwicha*, and fruits; unhealthy foods not pictured included high-sugar processed foods such as cookies and candy.

In examining responses from both semi-structured interviews and pile sort data, a number of influences on eating at the individual, social environmental, physical environmental, and macrosystem levels were identified. Key influences with exemplifying quotes are highlighted in Table 1.

**Table 1:** Key Influences on Eating in Adolescents Ages 15–17 years (*n* = 14) in Periurban Lima, Peru

Influence Identified	Exemplifying Quote
Individual	‘My friends don’t want to eat because they want to stay in shape; they don’t want to eat beans because they make them gain weight.’ ‘Some friends don’t eat well because they don’t have a family, home, or money.’ ‘Some friends don’t have money or they are alone, depressed and don’t want to eat.’
Social environmental	‘My grandmother and my mother tell me what is good for my health; I should stop eating the junk food that I buy on the street and eat fruit. I shouldn’t drink a lot of soda, and should drink water instead.’ ‘During break at school, my classmates and I share a liter of soda and crackers.’
Physical environmental	‘Sometimes I bring fruit from home for the school break, and sometimes I buy chips.’ ‘I buy my snack at school; there is a lady who prepares things like chicken with rice, fried rice, chicken soup, apple juice, oatmeal, and hot chocolate with milk.’ ‘In the afternoon, when I’m out, I buy things like crackers, chips and soda.’
Macrosystem	‘I looked up information about a food I had never seen in Wikipedia. I believed the information, as Wikipedia is a credible source.’ ‘I used Google to find information about bulimia, anorexia, and the food pyramid. With anorexia, you become thin and die little by little; it is contagious. I believed this because it was the same information my teacher presented.’

### Individual influences

#### Concerns about body image

Participants, particularly females, mentioned concerns about body image and desire to appear ‘thin’ as affecting food choices. When asked about barriers to eating well, one female participant said that some may not eat well because “they don’t like certain foods, or they say ‘this food will make me fat.’” Starchy foods such as bread, as well as beans, were most commonly cited as foods that may cause weight gain.

### Financial resources

Participants also mentioned lack of financial resources to purchase food, particularly when asked about barriers to healthy eating. When describing what factors may prevent his peers from eating well, one male participant said, “Maybe they don’t have enough money to be able to eat well, or maybe their father leaves them money to be able to cook and they spend it on other things, like games.”

### Nutrition-related knowledge

Participants demonstrated knowledge both with regards to eating for maintenance of health and prevention of diet-related conditions, which may potentially influence behavior. Participants’ description of ‘eating well for health’ reflected their familiarity with the concepts of moderation and balance. One participant described eating well as “not eating too much,” and another cited the importance of “eating a balanced diet—not eating a lot of fat, starch, or sweets.” There was also mention of consumption of unprocessed foods; one participant said that healthy eating means “eating good, natural food, and very little processed or canned food.” Some participants also mentioned the nutritional value of specific foods. As one participant put it, “healthy eating means eating dishes that have ingredients with vitamins, like fish, fruit, beans and vegetables.” When asked about prevention of anemia, participants named foods such as beans and fish as being important in the diet. Others had less knowledge of valuable foods for anemia prevention, and mentioned the importance of the consumption of grains such as wheat and kiwicha, while others cited non-dietary factors such as sleep and exercise as being most important.

### Taste

In both the pile sort exercise and in interviews, participants identified the foods they perceived to be ‘tasty.’ ‘Junk’ foods such as the Peruvian soft drink Inca kola (Coca-Cola Company, Atlanta, GA, USA) and other sugar-sweetened beverages, crackers, and chocolate were often identified as foods pleasing to the taste but also harmful in excess, a ‘waste of money,’ and ‘non-nutritive.’ When asked about their favorite food and drink in interviews, many participants identified the seafood dish *ceviche* and soft drinks as their preferences. In the pile sort activity, when asked to name ‘healthy’ foods that they considered to be tasty, participants most often identified foods such as fruit, beans, milk and meat.

### Social environmental influences

#### Family

Participants reported that their families’ guidance helped them to make healthy food choices; parents provided advice on food selection and fostered cooking skills. Parental advice was related to consumption of foods such as fruit, vegetables, and dairy products. In addition, parents advised participants to consume a variety of foods and to limit LNED foods such as sugar-sweetened beverages. Family members also stressed the importance of healthy, regular meals for disease prevention, and encouraged adolescents to consume foods prepared at home rather than purchased from street vendors. Adolescents reported helping their mothers to prepare dishes served at home, and in many cases were able to list the ingredients and quantities in recipes used.

Participants’ families also provided home-cooked meals. The majority of participants reported consuming all meals at home, with the exception of foods consumed during breaks at school. Most participants also reported eating meals in the presence of family members, most often their mothers and siblings. Both adolescents and their mothers were responsible for serving food prepared at home; most participants stated that their mother served them on at least some occasions at mealtime.

### Peers

Comments revealed potential peer influences on eating. With peers, participants reported sharing LNED items such as sugar-sweetened beverages and packaged snacks, particularly during breaks at school. In some cases, a large bottle of soda was purchased to be shared among friends. Comments also revealed potential peer influences on desired body size. When describing barriers to healthy eating, one participant said, “Some of my friends don’t eat well because they don’t have enough money or because they want to look thin. My classmates only eat salad at lunch.”

### Physical environmental influences

#### School

Many participants reported consumption of snack foods during the break at school. Foods available for purchase were generally LNED items such as sugar-sweetened beverages and packaged snacks. In some cases, prepared food such as chicken soup and fried rice was also available for purchase during the break. Participants who reported bringing their own snack to school rather than purchasing more often consumed items such as fruit.

### Fast-food establishments

The majority of participants reported consumption of fast food, such as fried chicken, French fries with sausage, hamburgers, and pizza. These foods were easily available for purchase and in some cases were taken home to eat. Among those who reported intake of fast food, frequency of consumption varied, with some participants reporting that they more commonly consumed these items on the weekend days, and others reporting daily consumption. When describing her weekend activities, one female participant said, “On the weekend I stay at home, help my mother, watch television, and I buy French fries with sausage.”

### Convenience stores and street vendors

A number of products, particularly snack foods, were purchased from stores in the community and street vendors. Participants reported purchasing items such as popsicles, toasted corn, chocolate, and soda. Similar to fast food, some participants mentioned that they more commonly consumed these items on the weekend days.

### Macrosystem influences

#### Media

When asked to describe their daily routines, all but three participants mentioned the television and/or Internet as part of their schedule. Several participants mentioned that they had heard information about nutrition and health on television. The messages ranged in topic from health benefits of specific foods, to food safety, to limiting food waste.

Nearly all participants said that computers interested them and that they used the Internet frequently for both schoolwork and social activities. One female participate noted, “I like to learn new things from the Internet, and play games. I use Messenger and Facebook.” Another male participant said, “I go with my friends after school. It’s fun to search for information, play games and watch movies and anime [Japanese animated productions].” All except one participant had used the Internet search for nutrition information and viewed Internet sources as credible. One female participant said, “I looked for information about a balanced diet. I found out that fruit contains vitamins, and the foods that make up a balanced diet are meat, chicken, eggs, milk and beans. I used Google, and believed this information because my teacher said it was right.”

## Discussion

To maximize the chance of success of nutrition education interventions, it is critical to consider the multitude of influences on adolescent eating habits. The current study employs a multi-method approach to provide insight into the factors that may have an impact on behaviors of adolescents in periurban Lima, Peru. Study findings may guide future research seeking to examine the influence of specific factors identified to inform programs aimed at improving dietary intake in youth.

Family was consistently described as having positive influence on food habits and skills. Previous research in diverse populations of adolescents suggests that family meals may have benefits in terms of dietary intake, disordered eating behaviors, substance use and psychosocial health [42, 43]. Other studies in the U.S. examining associations between family meal frequency during adolescence and diet quality, meal frequency, social eating, and meal structure later in life indicate that family meals during adolescence may have a lasting positive influence on dietary quality and meal patterns in young adulthood [44, 45]. Previous research in the U.S. has also shown that the protective effect of family meals may be attributed to interpersonal factors, such as healthy communication and parental positive reinforcement [46, 47]. Supporting family relationships as promising avenues to healthy eating may be key in improving dietary intake; however, studies are needed to further examine the relationship between familial factors and behaviors in Peruvian adolescents. Of note, a recent study in Colombian children and adolescents found that being part of an extended family predicted the likelihood of a child being overweight or obese [48]. The authors note that extended family members may influence the behavior of youth, so that having someone such as a grandmother as a main caregiver may increase the likelihood of overweight [48]. Future studies in the Peruvian population may similarly involve examining the relationship between family structure and bonds and eating habits. Barriers and facilitators with regards to family meals may be examined. Data may also be collected from parents and other caregivers to determine how they view their role in promotion of healthy eating, provision of main meals at home, and the degree to which they serve as role models for adolescents. A recent study conducted in Peruvian children points to the importance of examining parental modeling in this population, as interviews with parents revealed that fathers or husbands sometimes brought home soda, and two mothers were “not able to live without soda” [49]. Additional studies on the influence of parents on the behavior of youth may inform interventions that target whole families as a means of improving dietary intake of adolescents.

Potential peer influences on eating habits were also identified. Adolescents reported consuming LNED items when they were with their friends. A recent review of the literature indicates that peer influences on eating are widespread, and that in the presence of peers and friends, children and adolescents’ energy intake increases [11]. Such results have been found in adolescents in the U.S., with the presence of friends contributing to increased food intake in youth [50]. Previous research in Australia has also suggested that adolescents’ friends influence their intake of LNED foods over time [51]. In Peruvian adolescents, it may be important not only to consider the role of peers in promoting intake of certain foods, but also in addressing food insecurity. In a study exploring the lives of Peruvian adolescents in a low-income area outside of Lima, Bayer et al. report one adolescent’s account of giving food to a friend who lacked economic resources [52]. Future studies may seek to elucidate the mechanisms of social influence on behaviors of Peruvian adolescents and to determine how peers may favorably or unfavorably influence intake, and whether there are differences in the influence of peers in male versus female social groups. In addressing unfavorable behaviors that may result from peer influence, training peer leaders to model and promote healthy behaviors through teaching other youth how to change behavior may be an effective strategy [53, 54]. A recent study in adolescents in the U.S. focused on obesity prevention in the school setting through peer advocacy of healthy eating suggests that interventions should actively involve all students in advocacy to promote healthy behaviors [55].

In terms of macrosystem influences, the current study revealed that adolescents had frequent exposure to the television and Internet. A recent study also noted the large amount of television watching in Peruvian youth, with children watching as early as when they wake up in the morning, then during lunchtime (after returning from school), and then again after completing their homework from 5 to 9 pm or 10 pm [49]. Adolescents in the current study made use of the television and Internet for a variety of purposes. Among these purposes was to obtain information related to nutrition, which adolescents uniformly viewed as credible. In designing nutrition education interventions, it is important to consider not only the frequent use of technology, but also the degree to which adolescents trust the information gleaned from sources such as the television and Internet. To reach this demographic group, computers and mobile phones may be used as vehicles for information delivery. A recent systematic review of mobile-phone based interventions for health did not identify any studies conducted in Latin America, revealing the paucity of research in this area [56]. Technology may also be useful for data collection in interventions, as adolescents are adept in using electronic devices. The ‘mobile food record’ is a new tool that may hold promise for dietary data collection in this population; this tool has been tested in adolescent populations in the U.S. with positive results [57, 58]. The role of the rapidly changing electronic information environment in influencing youth knowledge and health behavior is also important for nutrition educators to note, especially given the attractive and novel ways technologies can deliver messages.

Since adolescents in Peru are more connected today through channels such as the Internet than they were in previous years [59], the influence of media images and messages on beliefs and behavior should also be considered. In providing comments on various food items in the pile sort activity, participants described meat as “fatty” and “containing hormones.” As there is information readily available on the fat in meat and its negative effects on the Internet and in newspapers and magazines, it is possible that these views were spurred by media messages. A number of studies in the U.S. [60–63], Canada [64], and Europe [65] have demonstrated that health information has a significant impact on consumer behavior. The degree to which the media may influence Peruvian adolescents’ views of specific foods as well as their eating habits should be examined in greater depth.

In addition, the potential media influences on the perception of ideal body weight may be further explored. Several participants mentioned that maintaining a low body weight was a priority among their peer groups. Previous studies have identified the media as one of the main purveyors of messages about the ideal body to youth, with thinness promoted in girls, and a muscular body considered ideal in boys [66–68]. While the media has been found to have a strong influence on body image perceptions of adolescents’ in Western nations [69], however, more research is needed to determine whether it may have a similar influence on diverse populations of adolescents in Latin America. In a study exploring the attitudinal and perceptual dimensions of body image among adolescents in six countries in Latin America, the authors speculated that frequent exposure to media-portrayed images of slender female bodies may have led to the strong appreciation for the thin female body observed in the study [70]. A study of Chilean adolescents, however, revealed that although girls reported higher perceived pressure from the media to lose weight than their male counterparts, media influences did not significantly contribute to their weight loss behaviors [71]. Additional studies to determine the effects of media on behavior in Peruvian adolescents are warranted. If the media is found to have a significant influence on body image and weight loss behaviors in this population, implementation of programs seeking to promote media literacy, such as the interactive wellness program “In Favor of Myself” recently conducted in Israel [72], may be explored.

Of note, participants often reported computer use as a social activity, and often went to Internet cafes with friends to listen to music, chat or play games. Some cited the use of social media sites such as Facebook during their time online. The social nature of Internet use in adolescents may have important implications for development of interventions to address nutrition-related concerns. In delivering nutrition education messages, development of multi-player games may be an attractive option; interactive activities may also be incorporated, such as discussion groups with peers. Social media may also prove to be a useful tool in conveying information. A recent review of school-based Internet obesity prevention programs for adolescents in the U.S. and Europe indicated the majority were successful in reaching high risk students and changing behaviors in the short-term [73]; future studies may investigate the usefulness of such programs in the Peruvian population.

In addition to the influence of media on behavior, nutrition education messages conveyed in school or elsewhere may also have an impact. Adolescents’ groupings of foods in the pile sorts activity reflected current educational messages presented to school-age children, while comments during the activity revealed a host of factors affecting food choices. One current nutrition-related resource available to school-aged children is the *discolonchera escolar*, a disc-shaped graphic accompanied by a manual that presents examples of healthy lunches and an explanation of the health benefits of each of the food groups [74]. While participants often grouped foods based on ‘type’, such as grains or vegetables, as presented in the *discolonchera escolar,* they also categorized foods based on likes/dislikes, frequency of consumption, eating occasion, taste, price and effect on health. In describing the categories, the concept of moderate consumption emerged as well as recommended consumption of foods such as fruit, vegetables, beans and whole grains. The nutrition education messages to which adolescents are exposed may affect food choices, along with a host of other factors at each of the four levels of influence on behavior.

### Limitations

Limitations of the current study include the individual unit of analysis examined in data collection. Through examination of perspectives of other individuals playing a role in influencing adolescent behaviors, such as parents, a clearer picture of the influences beyond the individual level may have emerged. There was limited mention of several issues that may have potentially important impacts on behavior, such as eating disorders. It may have been useful to address such issues more specifically in the interview guide, and to have allowed more time for an in-depth discussion of such topics. Other techniques, such as focus groups, could also potentially be used to gather more detailed information on pertinent issues, such as peer influences. Qualitative studies using focus groups in other adolescent populations have explored meanings of thinness, dysfunctional eating, and body dissatisfaction, with implications for prevention of eating disorders, which are common in this age group [75, 76]. Future studies in Peruvian adolescents might involve collection of more in-depth data on additional behavioral influences on eating of importance identified in these preliminary studies and other data collection methods such as focus groups. A future study may also further examine influences on physical activity, an important health-related behavior. Due to time constraints in interviews, limited questions were posed on this topic and results are not reported. An additional limitation was that participants were recruited from only one periurban region in Peru. These large periurban areas are home to about one third of the population of Lima of 8 million inhabitants, and most have expanded through migration from rural areas [77]. Nevertheless, the restriction to one community in one of the periurban districts inevitably yields limited diversity among the respondents, and it is possible that some of the views of the participants were specific to the area under study. Despite these limitations, this study did allow for identification of key socio-cultural influences on behavior in an understudied group of adolescents, with implications for development of programs to address nutrition-related issues in Peruvian youth.

## Conclusions

Further research is warranted to provide more detail on the roles of the factors identified in this research that shape behavior, particularly that of family. Future studies may involve examining the relationship between family structure and bonds and eating habits. Addressing nutrition-related issues such as obesity and iron-deficiency anemia in this population requires consideration of the effect of social and environmental factors in the context of adolescent lifestyles on behavior. Nutrition education messages for adolescents should consider the cultural perceptions and importance of particular foods, taking into account the diverse factors that influence eating behaviors.

## Abbreviation

LNED: Low-nutrient energy-dense
